# Editorial for the Special Issue on Advanced Manufacturing Technology and Systems, 3rd Edition

**DOI:** 10.3390/mi17070861

**Published:** 2026-07-21

**Authors:** Youqiang Xing, Guochao Li, Zhaoju Zhu

**Affiliations:** 1 School of Mechanical Engineering, Southeast University, Nanjing 211189, China; 2 School of Mechanical Engineering, Jiangsu University of Science and Technology, Zhenjiang 212100, China; liguochao@just.edu.cn; 3 School of Mechanical Engineering and Automation, Fuzhou University, Fuzhou 350108, China; zhaojuz@fzu.edu.cn

Advanced manufacturing technology and systems (AMTS) combine principles of mechanical engineering with design innovation to produce high-quality products with enhanced efficiency, flexibility, and precision. AMTS encompasses a wide range of innovative technologies, including additive manufacturing, precision manufacturing, robotics, automation, computer-integrated manufacturing, smart and flexible manufacturing, system optimization, etc. [1,2]. They enable smarter production by reducing waste and lowering energy consumption, as well as shortening product development cycles. Widely adopted in industries such as aerospace [3], automotive [4], electronics [5], and medical devices [6], AMTS play a critical role in advanced and sustainable manufacturing. This Special Issue presents the latest developments in AMTS.

This Special Issue contains 20 original papers about AMTS. Specifically, many research fields are covered: advanced manufacturing technology (nine papers), intelligent equipment design and control (six papers), surface engineering and performance optimization (three papers), and review (two papers). These studies are briefly summarized as follows.

Advanced manufacturing technology: This part mainly contains traditional cutting processing methods (Contributions 1, 9, 12 and 14) and non-traditional processing methods (Contributions 4, 7, 8, 13 and 18). The relevant contents are summarized as follows. Traditional cutting processing: Song et al. (Contribution 1) developed a precise cutting force model for discrete-edge end mills based on an effective chip slot function. Experiments exhibited notable advantages in machining stability, cutting force control, and surface quality. To improve surface quality and tool life in conventional machining of AerMet100 steel, laser-assisted machining (LAM) turning was developed by Tang et al. (Contribution 9). A theoretical model for surface formation in cutting-speed-direction ultrasonic-assisted turning (CUAT), covering both continuous and intermittent regimes, was proposed by Nguyen et al. (Contribution 12). A milling chatter stability analysis method based on the localized differential quadrature method (LDQM) was proposed by Mei et al. (Contribution 14). Non-traditional processing: Zhang et al. (Contribution 4) optimized magnetic abrasive polishing (MAF) for MP35N alloy stent inner walls using plasma-fused Al_2_O_3_ abrasives. Zheng et al. (Contribution 7) developed a structured-laser profile extraction method for wire arc additive manufacturing of inclined thick-walled structures. A friction stir processing was proposed to fabricate medium-Mn steel, resulting in hetero-deformation-induced and dislocation strengthening (Yang et al., contribution 8). A thermal reflow and ICP etching process for large-area silicon microlens arrays was proposed by Wu et al. (Contribution 13). Tang et al. (Contribution 18) studied graphite-powder-mixed electrochemical discharge machining of glass microholes, improving surface quality, mechanical properties, and processing efficiency.

Intelligent equipment design and control: Zhang et al. (Contribution 2) designed a two-stage reduction micro-drive mechanism based on the particle swarm algorithm and optimized its structure via the particle swarm algorithm, achieving excellent dynamic performance and a large reduction ratio. Yang et al. (Contribution 3) developed a dynamic continuous error compensation model for direct-drive turntables based on a “decomposition-modeling-integration-correction” strategy, reducing the positioning error standard deviations. A fully automated collaborative heterogeneous mini-robotic 3D food printer with adaptive control and screw conveyor configuration, enabling the fabrication of complex multi-material food structures, was proposed by Mendoza-Bautista et al. (Contribution 5). A near-field direct writing (NFDW) technique incorporating piezoelectric micromotion control with macroscopic movement was developed for precise fabrication of serpentine micro-/nanofibers (Chen et al., contribution 6). A strain elastic element with a double-layer cross-floating beam for wireless rotating dynamometers was proposed, which had high sensitivity and low cross-sensitivity errors (Wang et al., contribution 10). Fang et al. (Contribution 17) investigated intelligent compliance control strategies for robotic ultrasonic strengthening of aviation blade surfaces.

Surface engineering and performance optimization: Wang et al. (Contribution 11) synthesized spherical Al_2_O_3_ magnetic abrasives via plasma molten metal powder and powder jetting, achieving improved surface roughness on AZ31B alloy through magnetic abrasive finishing. Zhu et al. (Contribution 15) conducted numerical analysis of stress force on vessel walls during coronary rotational atherectomy using computational fluid dynamics, revealing that smaller burr-to-artery diameter ratios (B/A = 0.5) result in more stable flow fields. Wu et al. (Contribution 16) demonstrated that the combination of laser surface texturing and MoS_2_ solid lubricants effectively reduces the friction coefficient and adhesion of Ti-6Al-4V alloys.

Finally, the exploration of the cutting processing mode of low-rigidity parts (Zhu et al., contribution 19) and research progress of laser-processing technology in diamond micro-fabrication (Zhang et al., contribution 20) were reviewed.
